# Thyroid nodules in childhood: indications for biopsy and surgery

**DOI:** 10.1186/1824-7288-40-48

**Published:** 2014-05-19

**Authors:** Filippo De Luca, Tommaso Aversa, Luca Alessi, Valeria Cama, Daria Costanzo, Cristina Genovese, Veronica Scuderi, Roberta Vadalà, Giuseppe Zoccali

**Affiliations:** 1 Department of Pediatrics, University of Messina, Via Consolare Valeria, 98124 Messina, Italy

**Keywords:** Fine-needle aspiration biopsy, Cancer predictive value, Malignancy predictive signs, Risk factors, Thyroidectomy

## Abstract

Aims of this commentary is to report the most recent views about epidemiology, diagnostic procedures, malignancy risk factors and clinical management of thyroid nodules in children. On the basis of our personal experiences and recent literature evidences, we conclude that: a) if nodule is accompanied by lymphadenopathy and/or other alert findings, fine-needle aspiration biopsy (FNAB) should be recommended; b) if no lymphadenopathy and no other clinical and ultrasonographic alert signs are observed, work-up can progress to FNAB only if nodule persists or grows over time, even under levo-thyroxine therapy.

## Background

Thyroid cancer, i.e. the fifth leading cancer in women, the most common endocrine malignancy and also the fastest rising cancer in both genders [1,2], usually presents as a thyroid nodule. Although nodules in childhood are rare, they are not infrequently malignant (up to 25% of cases) and therefore require a careful evaluation and an aggressive diagnostic approach.

Due to the above considerations, we believe that this issue deserves a specific commentary, aiming to report the most recent views about epidemiology, diagnostic procedures, malignancy risk factors and clinical management of nodular disease in childhood.

### Epidemiology

Nodule prevalence in adults has been estimate to range from 2 to 6% by palpation, from 19 to 35% by ultrasonography (US) and from 8 to 65% in postmortem examinations [3]. Although the epidemiological studies in pediatric age are very scanty, nevertheless US prevalences in childhood may be estimated to range from 0.2 to 5.1% [4,5]. However, the cancer risk of a thyroid nodule is significantly greater in children than in adults (22 vs 14%) [6]. Even rates of cancer metastasis and extrathyroidal extension have been found to be much higher in children [7,8].

Also sex distribution seems to change with age. In fact, whereas in adults women outnumber men 4:1, in children below 15 the ratio of girls to boys is 1.5:1 and in patients aged 15–20 the female: male ratio is 3:1 [9].

A significant risk factor for the occurrence of thyroid nodular disease is the association with Hashimoto’s thyroiditis (HT), as suggested by a recent study demonstrating an increase prevalence of nodules (31.5%) in children with HT [10].

### US and clinical risk factors for thyroid cancer

Excluding malignancy is the diagnostic goal of thyroid nodule evaluation in both adults and children [11].

In children a nodule should be reviewed with suspicion, due to the relevant relative risk of malignancy in pediatric age [4]. In particular, it has to be considered, on the basis of epidemiological data, that males and very young children (<10 years) are the patients with the highest risk of cancer [4].

Cancer rate is significantly associated with method of nodule discovery and the patients with ultrasonographic (US) incidentalomas have the lowest malignancy rate (4%), whereas those with clinical diagnosis have a much higher cancer prevalence (30%) [12].

Although a thyroid US cannot clearly discriminate between benign and malignant nodules, it does provide very useful information in selecting the children to be evaluated by fine-needle aspiration biopsy (FNAB). Among US parameters, hypoechogenicity, microcalcifications, specific lymph node alterations and increased intranodular vascular flow are all findings indicating a high suspicion for cancer [13].

The lymph node US alterations which have been found to correlate with an increased risk of malignancy are: irregular margins, heterogeneous echoic pattern, calcifications and irregular vascularity throughout the lymph node instead of the normal central hilar vessels at Doppler imaging [14].

The anamnestic and clinical findings that are considered to have some predictive value for malignancy are: family history for thyroid cancer, previous oncohematological disorders, history of irradiation exposure, presence of palpable lymph nodes and symptoms attributable to compression of adjacent structures (discomfort, dysfagia, dysphonia, hoarseness, breathing obstruction) [14]. From a clinical point of view, another aspect which should be considered in the evaluation of nodule prognosis is whether isolated and multiple nodules have the same risk of malignancy. Consistent with other reports [15], our results suggest that cancer may also be present in multinodular thyroid disease, although the majority of malignant nodules are isolated [14].

Finally, spontaneous growth of nodules and effectiveness of levo-thyroxine (L-T4) therapy in shrinking their dimensions may give further information in the evaluation of nodule prognosis. In fact, a significant size reduction (>50%) is known to occur in 30% of children with benign nodules, whereas in untreated subjects both palpable nodules and symptoms increase [16].

### Other diagnostic investigations

Among the conventional diagnostic investigations, FNAB is, even in childhood and adolescence, the one that offers the best sensitivity (95%), specificity (86%), and accuracy (90%) rates in detecting malignancy [17]. These inferences have been more recently confirmed [14,18,19] and, therefore, it can be argued that FNAB plays a crucial role in the management of children’ thyroid nodules, since it allows a selection of patients to undergo surgery [14].

Measuring thyrotropin (TSH) and free thyroxine levels at nodule detection is mandatory to assess thyroid function and establish the further work-up. Patients with hyperthyroidism are likely to be affected by toxic adenomas and, therefore, scintiscan is indicated. However, the large majority of children with either benign or malignant nodules are euthyroid and only a small fraction of thyroid cancers can present with hot/warm scintiscan uptake [14].

Blood calcitonin level measurement may be very useful at nodule detection, because this test is crucial for an early diagnosis of medullary carcinoma.

Among thyroid function tests, TSH measurement seems to be possibly used also for a prognostic purpose, since the highest TSH quintiles have been just recently demonstrated to be associated with an increased risk of malignancy [20].

### Relative histotypes of pediatric thyroid nodules

According to the results of a recent paper of our multicenter study group, malignant histotypes were found in 30% of children with nodular disease [14]. The most frequent malignant histotype was papillary carcinoma (22.2%), followed by follicular carcinoma (4.7%) and medullary carcinoma (3.2%), whereas the most frequent benign histotype was goitrous nodule (53.4%) followed by follicular adenoma (12.7%), Hurthle cell adenoma (3.2%) and benign teratoma (1.6%). All the patients with a malignant histotype had exhibited at cytologic evaluation an either malignant or suspicious aspect, with no false negatives. Based on these results, FNAB had 100% sensitivity, 83.3% specificity and 89.1% diagnostic accuracy [14].

### Pre-surgical management of children’ thyroid nodules

Even though the long-term prognosis of children with thyroid malignancy may be favourable, even in the cases with extrathyroidal extension and metastasis [7,8], early detection improves outcome [15]. Moreover, many children with thyroid nodules undergo extensive and costly work-up before being referred to surgeons [21]. Due to these reasons it is mandatory to identify the minimal work-up preceding surgery.

On the basis of our personal experiences [10,14,16,17,20] and recent literature evidences [6,12,13,21], we suggest that: a) if a palpable nodule in a euthyroid child is accompanied by lymphadenopathy or signs of compression and/or other alert findings (Table 1), the suspicion of malignancy may be strong and FNAB should be recommended; b) if no lymphadenopathy and no other clinical and US alert signs are observed, work-up can progress to FNAB only if nodule persists or grows over time, even under L-T4 therapy.

**Table 1:** Anamnestic, clinical, laboratory and ultrasonographic (US) risk factors for malignancy in the diagnostic approach to children’ thyroid nodules

**Anamnestic data**	**Laboratory data**
– Family history for thyroid cancer	– Increased thyrotropin levels
– Previous cancer	– High calcitonin levels
– History of irradiation exposure	**US data**
**Clinical data**	– Nodule hypoechogenicity
– Lymphoadenopathy	– Nodule microcalcifications
– Signs of compression of adjacent structures	– Nodule undefined margins
– Nodule growth under levothyroxine therapy	– Increased intranodular vascular flow
	– Specific lymph node alterations

### Follow-up of children with benign thyroid nodules on FNAB

The initial finding of a benign lesion on FNAB does not exclude per se the presence of a malignancy, since cytological evaluation has a sensitivity which ranges from 75 to 95% in the different studies [14,17-19,22]. Therefore, it advisable that pediatric endocrinologists continue to follow over time their young patients with persisting nodules, even those with previously documented benign lesions on FNAB. Moreover, it has to be considered that persisting palpable/ visible nodules proven to be benign by FNAB may still be causing long-lasting worries for the children’s families.

Follow-up of young patients with initially detected benign nodules should take into consideration all the above described clinical and US predictive factors of malignancy. In particular, the factors that have been reported to be most frequently associated with cancer risk are: family history of thyroid cancer, hypoechoic lesions and palpable lymph nodes [23]. In children with persisting nodules, the presence of any of these risk factors should heighten suspicion for malignancy, even in the setting of a benign FNAB [23].

It is sometimes discussed, among the pediatricians, whether the long-lasting anxieties of children’s parents for the persistence of a nodule would be an indication for surgery. According to other Authors, patients with a benign FNAB and no clinical and US criteria for malignancy may not need to be referred to the surgeon [23]. In our opinion, such inference might be largely subscribed to.

## Conclusions

a) thyroid nodules in childhood are not infrequently malignant and require a well-established diagnostic approach; b) the children to be evaluated by FNAB should be selected by analysis of clinical and US risk factors; c) lymphadenopathy and compression signs are the most specific clinical signs; d) nodular microcalcifications and specific lymph node alterations are the most reliable US risk factors; e) in the cases with either positive or suspicious cytological evaluation after FNAB, surgery has to be strongly recommended; f) in the patients with benign thyroid nodules on FNAB clinical follow-up with serial physical and US examinations is recommended; g) surgery might be taken into consideration even in the cases with previously documented benign lesion on FNAB, when the strongest clinical and US risk factors are present; h) the most frequent malignant histotype is papillary carcinoma.

## Abbreviations

FNAB: Fine-needle aspiration biopsy; HT: Hashimoto’s thyroiditis; L-T4: levothyroxine; TSH: Thyrotropin; US: Ultrasonography.

## Competing interests

The authors declare that they have no competing interests.

## Authors’ contributions

FDL has given final approval of the version to be published; TA has been involved in revising the manuscript for important intellectual outcome; LA, VC and GZ have given substantial contributions to conception and design; DC and CG have given substantial contribution to acquisition, analysis and interpretation of data; VS and RV have been involve in drafting the manuscript and looking for the most suitable references. All authors read and approved the final manuscript.
